# Improvement of Histamine Intolerance Symptoms in Pregnant Women with Diamine Oxidase Deficiency: An Exploratory Study

**DOI:** 10.3390/jcm14134573

**Published:** 2025-06-27

**Authors:** Adriana Duelo, Sònia Sánchez-Pérez, Salvador Pellicer-Roca, Sara Sánchez-Buxens, Oriol Comas-Basté, M. Luz Latorre-Moratalla, M. Carmen Vidal-Carou

**Affiliations:** 1 Departament de Nutrició, Ciències de l’Alimentació i Gastronomia, Campus de l’Alimentació de Torribera, Universitat de Barcelona (UB), 08921 Santa Coloma de Gramenet, Spain; aduelo@ub.edu (A.D.); spellicer@ub.edu (S.P.-R.); oriolcomas@ub.edu (O.C.-B.); mcvidal@ub.edu (M.C.V.-C.); 2 Institut de Recerca en Nutrició i Seguretat Alimentària (INSA·UB), Universitat de Barcelona (UB), Av. Prat de la Riba 171, 08921 Santa Coloma de Gramenet, Spain; soniasanchezperez@ub.edu; 3 International Institute of DAO Deficiency, C/Escoles Pies 49, 08017 Barcelona, Spain; sara@icddao.com; 4 Institut d’Investigacions Biomèdiques August Pi i Sunyer (IDIBAPS), c/Rosselló 149, 08036 Barcelona, Spain

**Keywords:** histamine intolerance, diamine oxidase (DAO) enzyme, DAO deficiency, women’s health, pregnancy, histamine intolerance symptoms

## Abstract

**Background/Objectives**: Diamine oxidase (DAO) deficiency can lead to excessive histamine absorption at the intestinal level, triggering symptoms that affect the gastrointestinal, neurological, dermatological, respiratory, circulatory, and musculoskeletal systems. This condition, known as histamine intolerance, is more prevalent in women. While serum DAO levels have been observed to increase during pregnancy in healthy women, there is a lack of in-depth studies evaluating the relationship between pregnancy, DAO activity, and histamine intolerance symptoms. This is the first study to assess serum DAO activity before, during, and after pregnancy, as well as the evolution of histamine intolerance symptoms in women diagnosed with this condition. Due to low histamine, diets are quite restrictive, no dietary intervention was considered for pregnant women. **Methods**: This prospective observational study used an assessment questionnaire to evaluate the presence or absence of histamine-related symptoms in 30 adult women with histamine intolerance before, during, and after pregnancy. Serum DAO activity was also measured at the three time points. **Results**: Nearly all women (27 out of 30) experienced symptom improvement during pregnancy (*p* < 0.001). Specifically, at least 77% of women reported a marked reduction in flatulence, bloating, headache, rhinorrhea, flushing, pruritus, hypotonia, or muscle pain. Concurrently, the DAO activity significantly increased 11-fold from the baseline, coinciding with symptom relief. At two months postpartum, symptoms tended to reappear, accompanied by a significant decrease in DAO activity in all participants. **Conclusions**: This first-of-its-kind observational study demonstrates an improvement in histamine intolerance symptoms and an increase in serum DAO activity during pregnancy. The pronounced symptom relief suggests that restrictive diets, such as low-histamine diets, may not be necessary during pregnancy. Further research is required to confirm these novel findings.

## 1. Introduction

Diamine oxidase (DAO) is an enzyme responsible for metabolizing dietary histamine in the gut and potentially histamine produced by the intestinal microbiota [1,2,3]. Consequently, individuals with DAO deficiency may absorb excessive amounts of histamine at the intestinal level, leading to its accumulation in the bloodstream and the onset of a wide range of symptoms. This condition, known as histamine intolerance, is notably more prevalent among women, particularly those who are middle-aged [1,4].

DAO deficiency can be genetic or acquired [4]. Several single-nucleotide polymorphisms (SNPs) in the DAO-encoding gene (*AOC1*), located on chromosome 7 (7q36.1), have been associated with reduced DAO activity, particularly variants rs10156191, rs1049742, rs1049793, and rs2052129 [5]. Research indicates that the degree of alteration (i.e., whether the variants are in a heterozygous or homozygous state) is more significant than the presence of one or more altered variants [5,6,7]. Furthermore, Duelo et al. (2024) reported that patients carrying TT alleles of the rs2052129 variant, located in the gene promoter region, showed significantly lower serum DAO activity [5].

In addition to genetic factors, DAO deficiency can result from inflammatory conditions or impaired intestinal function, which may restrict DAO secretion. It can also be induced by certain drugs known to inhibit DAO activity, including commonly used medications such as acetylcysteine, clavulanic acid, metoclopramide, and verapamil [4]. In addition to these etiological factors, intestinal dysbiosis has been proposed as a potential contributor to DAO deficiency. Although research in this area remains limited, studies suggest that alterations in gut microbiota composition may reduce DAO activity or that an overabundance of histamine-secreting bacteria could lead to histamine accumulation in the gut [3]. Understanding the role of gut microbiota in histamine intolerance remains a key challenge in this field of research.

Histamine accumulation in the plasma can trigger symptoms across multiple organs and systems owing to the widespread distribution of histamine receptors throughout the body [4]. While gastrointestinal symptoms such as bloating and constipation are the most common, other frequently observed manifestations affect the skin, as well as the neurological, respiratory, circulatory, and musculoskeletal systems [5]. Notably, individuals with histamine intolerance often experience two or more concurrent symptoms in response to dietary intake of histamine. This multi-system involvement makes diagnosis and treatment particularly challenging.

Currently, the only evidence-based strategy for alleviating or preventing symptoms is adherence to a low-histamine diet, often supplemented with exogenous DAO to enhance histamine breakdown at the intestinal level [8,9,10,11,12,13,14,15,16]. However, while this dietary approach can improve symptoms, it does not appear to influence serum DAO activity [17]. Interestingly, some studies have reported an increase in DAO activity during pregnancy [18,19,20,21,22,23]. It has been suggested that the placenta enhances DAO production, acting as a metabolic barrier to prevent excessive histamine from entering the fetal-maternal circulation [24]. High levels of histamine in pregnant women have been linked to adverse outcomes, likely due to its vasoactive properties. Thus, maintaining a balance between histamine and DAO is crucial for a healthy pregnancy [21,23,24].

Serum DAO levels have been shown to increase during pregnancy, starting around the 12th week of gestation in healthy women [19,20,22,23]. However, in-depth research on the relationship between pregnancy, DAO activity, and symptoms of histamine intolerance remains limited. To address this gap, this study is the first to assess serum DAO activity before, during, and after pregnancy in women diagnosed with histamine intolerance while tracking the evolution of their symptoms. No dietary intervention was conducted during the study period, allowing the assessment to focus solely on the progression of DAO activity and changes in symptoms. Additionally, four genetic variants associated with DAO deficiency were identified in this cohort.

## 2. Materials and Methods

### 2.1. Participants and Study Design

An observational study was conducted with 30 women who presented with symptoms of histamine intolerance, recruited from a broader ongoing clinical trial. These women became pregnant between the eligibility assessment visit and the baseline visit (just before beginning the intervention) and thus left the main trial. They were then derived to a specialized center for dietary management of this condition (International Institute of DAO Deficiency—IDAO, Barcelona, Spain) to participate in this pregnancy-focused sub-study during 2022–2024. Participants with diagnosed allergies and/or those taking DAO-inhibitory drugs were excluded from the study.

Symptomatology and serum DAO activity were collected at three time points: before pregnancy, at 12–14 weeks of gestation, and two months postpartum. Data during pregnancy were collected between 12 and 14 weeks of gestation based on the limited literature [18,19,20,21,22,23]. Additionally, the prevalence of *AOC1* gene polymorphisms associated with DAO deficiency was assessed in this cohort. Four participants experienced miscarriages; therefore, no postpartum data were available for these cases.

All participants received detailed information about the purpose and procedures of the study and provided written informed consent for the use of their recorded data in this prospective study. This study was conducted in accordance with the Declaration of Helsinki of 1975. The broad clinical trial was approved by the Bioethics and Clinical Research Committee of the Hospital Clinic de Barcelona on 2 June 2022 (HCB/2022/0437), and this pregnancy-focused sub-study was approved by the Bioethics Committee of the University of Barcelona (IRB00003099) on 10 October 2024.

### 2.2. Recording Symptoms Associated with Histamine Intolerance

The type and frequency of symptoms in each participant were recorded by trained registered dietitians specializing in DAO deficiency at the IDAO through the administration of a prospective real-time questionnaire during each patient appointment. This tool qualitatively evaluated the presence or absence of 21 symptoms associated with histamine intolerance at three time points: before pregnancy, at 12–14 weeks of gestation, and two months postpartum. The symptoms recorded are presented in Table 1.

### 2.3. Determination of Serum DAO Activity

Serum DAO activity was analyzed using a radioextraction assay (DAO-REA (3H), Immundiagnostik AG, Bensheim, Germany) at the Echevarne Laboratory (Barcelona, Spain). This method measures the reaction product using radiolabeled putrescine dihydrochloride as a substrate. Radioactivity was quantified using a beta counter, with the signal directly proportional to the DAO activity in the sample. According to the parameters established by the Echevarne Laboratory, values below 12.54 U/mL were considered indicative of DAO deficiency. One U (unit) corresponds to the DAO activity required to degrade 1 μmol/mL of substrate per minute [9].

### 2.4. Identification of SNPs in the DAO-Encoding Gene (AOC1)

In this study, four SNPs (rs10156191, rs1049742, rs1049793, and rs2052129) were analyzed due to their reported association with DAO deficiency in Caucasian populations [5,6,7,25,26]. Briefly, genotyping of these DAO-encoding gene variants was performed using multiplex polymerase chain reaction (PCR) on the buffy coat fraction of blood samples obtained after centrifugation of blood collected in EDTA vacutainers. Real-time PCR was conducted using Open CFX^TM^ Manager software, version 3.1 (Bio-Rad^®^, Barcelona, Spain). The analysis was performed at Echevarne Laboratory (Barcelona, Spain).

### 2.5. Statistical Analysis

Statistical analyses were performed using SPSS Statistics 27.0 statistical software package (IBM Corporation, Armonk, NY, USA). Changes in the frequency of clinical manifestations during pregnancy were assessed using McNemar’s test. A paired-samples *t*-test was conducted to compare the mean number of symptoms at different time points. Pearson’s correlation was used to evaluate the relationship between DAO activity and the number of symptoms. Additionally, Friedman and Wilcoxon tests were used to assess changes in DAO activity during pregnancy. The association between *AOC1* gene variants and basal serum DAO values was analyzed using the Kruskal−Wallis test. Normality was assessed using Q-Q plots and the Shapiro−Wilk test. A *p*-value < 0.05 was considered statistically significant.

## 3. Results

### 3.1. Symptoms and DAO Activity in Participants Before Pregnancy

The baseline characteristics of the participants are shown in Table 1, including age, body mass index (BMI), DAO activity, and relevant clinical history. These data were presented to provide a descriptive overview of the demographic and clinical variables at the beginning of the study.

Symptomatology data collected from participants before pregnancy by registered dietitians using a specific questionnaire are presented in Table 2. On average, participants reported 6.9 ± 2.1 symptoms per individual, with 97% experiencing clinical manifestations in three or more organs or systems simultaneously. The gastrointestinal tract was the most commonly affected system, with 90% of participants reporting symptoms such as bloating, constipation, and flatulence. A significant proportion of patients also experienced diarrhea, reflux, and postprandial fullness. Neurological symptoms were highly prevalent, affecting 80% of the participants, with headache being the most frequently reported symptom, followed by migraine episodes. In the musculoskeletal domain, fatigue and muscle pain were the most common complaints among the participants. Dermatological symptoms, such as pruritus and flushing, were also frequently reported. Additionally, 43% of the participants experienced rhinorrhea and hypotension. No cases of asthma or tachycardia were observed (Table 2).

Serum DAO activity levels in patients prior to pregnancy ranged from 2.37 to 26.6 U/mL, with a mean value of 7.69 ± 5.2 U/mL. Notably, 87% of the women exhibited DAO levels below 12.54 U/mL, the threshold indicative of DAO deficiency according to this biomarker, suggesting impaired DAO enzymatic function in most of the cohort. Only four women had DAO levels above this threshold; however, they still reported a high number of histamine-related symptoms, even exceeding the mean of seven symptoms observed in the study population.

### 3.2. DAO-Encoding Gene Variants in Participants

Regarding the four specific *AOC1* gene polymorphisms associated with reduced DAO activity, 25 women (83% of the cohort) carried one or more altered SNPs. The frequency of each genotype and the corresponding allele frequencies for the SNP variants are detailed in Table 3. The genotyping data were in Hardy–Weinberg equilibrium, with allele frequencies consistent with those expected for the European population across wild-type, heterozygous, and homozygous individuals [27]. In this cohort, the rs10156191 variant was the most prevalent (65%), followed closely by rs1049793 (59%) and rs2052129 (52%) variants. When evaluating the allelic variant states (heterozygous or homozygous), heterozygous genotypes were more frequently observed.

This study found no statistically significant correlation between the number of genetic variants and the number or type of symptoms according to the Kruskal−Wallis test (*p* > 0.05). Additionally, symptomatology did not appear to depend on the allelic profile (homozygous or heterozygous).

Among the 25 women carrying at least one of the four *AOC1* gene variants, 21 exhibited DAO deficiency (<12.54 U/mL), as did all five non-carriers. Table 3 also presents the mean pre-pregnancy serum DAO activity according to genotype. Although DAO activity was low in all cases, a trend toward even lower activity was observed with a higher degree of allelic alteration for each SNP variant. However, the distribution of DAO activity values was statistically similar across genotypes, except for individuals carrying the rs1049793 variant in heterozygosity (CG), who had significantly lower DAO activity than those with the wild-type genotype (CC) (*p* = 0.021).

### 3.3. Symptoms in Participants During and After Pregnancy

**Figure 1:**
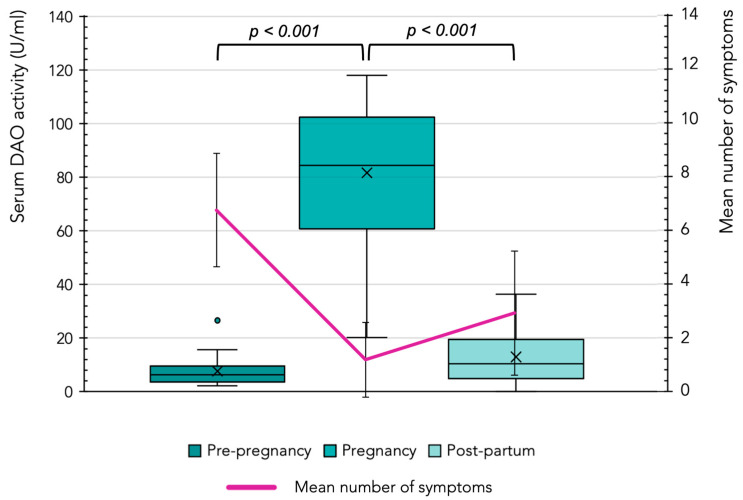
Serum DAO activity (U/mL) and mean number of symptoms (indicated by pink line) before, during, and after pregnancy. Serum DAO activity was compared using the Wilcoxon signed-rank test. Mean values are represented with an × and values statistically considered as outliers (atypical values) are plotted as circles.

Nearly all participants (27 out of 30) reported an improvement in symptoms during pregnancy, with a statistically significant reduction in the mean number of symptoms per individual, decreasing from 6.9 ± 2.1 to 1.38 ± 1.39 (*p* < 0.001) (Figure 1). As shown in Table 1, there was a marked reduction in the symptoms at the systemic level. Furthermore, the prevalence of most specific symptoms declined, with statistically significant differences observed in 14 symptoms compared to pre-pregnancy levels (*p* < 0.05). Gastrointestinal symptoms, which were the most frequently reported symptoms before pregnancy, improved significantly, with 49% of patients no longer experiencing these issues during gestation. Notably, flatulence and bloating decreased by 91% and 90%, respectively. Neurological symptoms also improved, with an 85% decrease in headaches in pregnant women. Additionally, there was a substantial reduction (at least 77%) in the prevalence of symptoms such as rhinorrhea, flushing, pruritus, hypotonia, and muscle pain. In contrast, only vomiting and tachycardia showed a slight increase in frequency compared to the pre-pregnancy period, both of which are commonly associated with pregnancy itself [28].

At two months postpartum, some symptoms that had declined during pregnancy tended to reappear. However, only the increase in migraine frequency was statistically significant (Table 2). The mean number of clinical manifestations per individual after pregnancy remained relatively low at 3.11 ± 2.3, although this was twice the mean observed during pregnancy (*p* = 0.002). Importantly, in no case did the number of symptoms return to pre-pregnancy levels within the two months postpartum period. Specifically, symptoms such as bloating, constipation, flatulence, rhinorrhea, and pruritus remained significantly lower than those before pregnancy. In contrast, vomiting, reflux, tachycardia, and lack of concentration, which are symptoms typically associated with pregnancy, did not reappear in individuals who had experienced them during gestation [29,30]. Data from four women were unavailable due to pregnancy loss occurring from the second trimester onward.

### 3.4. DAO Serum Activity in Participants During and After Pregnancy

Figure 1 illustrates the evolution of DAO activity (distribution of participant values) before, during, and after the pregnancy. A significant increase in DAO activity levels was observed in all women upon becoming pregnant, increasing from a mean of 7.69 ± 5.2 U/mL before pregnancy to 80.18 ± 26.8 U/mL (*p* < 0.001). At two months postpartum, the DAO activity had declined significantly in all women, with a mean value of 15.19 ± 9.7 U/mL (*p* < 0.001). However, in most cases, the DAO levels remained higher than the pre-pregnancy values. Additionally, Figure 1 overlays the evolution of the mean number of symptoms at the three time points, demonstrating that the increase in DAO activity coincided with a reduction in symptoms. Conversely, the postpartum decline in DAO activity corresponds to a mild recurrence of symptoms. Despite this trend, the Pearson correlation coefficients between changes in DAO activity and symptom evolution were low between pre-pregnancy and pregnancy (0.181) and between pregnancy and postpartum (−0.148). Interestingly, the increase in DAO levels during pregnancy was less pronounced in women who experienced spontaneous miscarriages, with mean values of 24.13 ± 5.78 U/mL compared to 86.9 ± 19.17 U/mL in the rest of the cohort.

## 4. Discussion

Some studies, albeit limited and outdated, have investigated serum DAO activity during pregnancy, primarily focusing on gestational complications associated with elevated histamine levels [19,20,21,22,23,31]. The present study is the first to assess serum DAO activity in pregnant women with histamine intolerance before and after childbirth and its relationship with symptom evolution.

In the current study, the mean number of symptoms reported by women before pregnancy was approximately 7, which is slightly lower than the 8 to 11 symptoms described in other studies of patients with histamine intolerance [5,32]. Nevertheless, the primary self-reported symptoms in our cohort align closely with those documented in previous studies, the most common of which were gastrointestinal, neurological, and dermatological symptoms (bloating, constipation, headache, migraine, pruritus, and flushing) [5,32,33]. Likewise, the DAO activity levels in our patients before pregnancy (7.69 U/mL ± 5.2) were comparable to those reported in numerous studies on histamine-intolerant individuals [9,17,34,35,36,37]. However, we did not observe a statistically significant correlation between a higher symptom frequency and lower serum DAO activity before pregnancy. Pinzer et al. (2017) also found no correlation between histamine intolerance symptoms and the measured DAO activity [33]. In contrast, Manzotti et al. (2016) and Cucca et al. (2022) reported a significant correlation between these variables [9,34]. Furthermore, Cucca et al. (2022) found that the symptom severity tended to decrease with higher DAO activity levels [34].

During pregnancy, the participants experienced a reduction in the frequency of nearly all symptoms, particularly those most commonly reported before pregnancy, such as bloating, flatulence, rhinorrhea, pruritus, flushing, headache, migraine, hypotonia, and muscle pain. Symptom improvement in histamine-intolerant patients with DAO deficiency has been previously described following the implementation of a low-histamine diet with or without DAO supplementation. However, most studies have focused primarily on dermatological symptoms, with only a few considering multiple physiological systems [10,11,16,34,36,38,39,40]. The women included in this study had either not yet started a low-histamine diet at the time of conception or, in most cases, had followed the diet for only a short period. A low-histamine diet is highly restrictive from a nutritional perspective (more than 30 foods are often excluded); therefore, it is not recommended during pregnancy [41]. Therefore, in this first exploratory study, no dietary intervention was implemented, and dietary intake was not recorded throughout the study period, making it impossible to evaluate the potential contribution of diet to the improvement of symptoms. However, during pregnancy, certain foods, many of which are potentially high in histamine, are generally avoided as part of the standard dietary recommendations. Thus, it is possible that some symptom improvements may have been partly influenced by these dietary restrictions. Moreover, the reduction in the number of symptoms coincided with a significant increase in serum DAO activity (from 7.7 to 87 U/mL). Several previous studies have also reported an increase in serum DAO levels during pregnancy in healthy women [18,19,20,21,22,23,31]. According to the literature, serum DAO activity begins to increase linearly from the early weeks of pregnancy and stabilizes during mid-gestation. For instance, Southren et al. (1966) and Carrington et al. (1972) reported that at 12–14 weeks, the mean DAO activity in uncomplicated pregnancies reached 150–300 U/mL, although with considerable variability among women [18,19]. Additionally, these authors documented that serum DAO levels continued to rise, reaching 1000–1500 U/mL at 20–22 weeks of gestation, and remained relatively constant thereafter until week 40. In this study, we were unable to assess DAO activity at weeks 20–22 and beyond, which limits our ability to capture the full trajectory of its increase during pregnancy. At 12–14 weeks, a high degree of inter-individual variability was also observed. One possible explanation for this variability is the sharp rise in DAO activity that appears to occur between weeks 12 and 22 of gestation, according to assessments performed by Southren et al. (1966) and Carrington et al. (1972). Since DAO activity in the current study was measured within a two-week time window (12–14 weeks), it is likely that participants were at slightly different points along the steep rising phase of the activity curve [18,19]. This timing difference may explain some of the inter-individual variability observed in DAO levels.

The increase in DAO activity during pregnancy has been interpreted as an adaptive mechanism to protect the fetus from excessive histamine exposure [18,19,20,21,22,23]. Holinka et al. (1984) demonstrated that progesterone administration leads to increased DAO activity in pregnant hamsters [42]. In humans, only one study has evaluated the influence of hormonal fluctuations on DAO activity during the menstrual cycle. In that study, an increase in progesterone levels during the luteal phase was accompanied by a corresponding increase in DAO activity, suggesting a potential hormonal influence on the DAO enzyme [43]. However, to the best of our knowledge, no human study has directly explored this relationship during pregnancy.

However, a notable finding was that the four women who experienced spontaneous miscarriage showed a smaller increase in DAO enzyme activity during pregnancy (20.2, 21.4, 23.2, and 30.8 U/mL) compared to the rest of the women, all of whom had DAO levels above 54 U/mL at weeks 12–14 of gestation. This observation aligns with the findings of Southren et al. (1966), who observed DAO activity levels below 50 U/mL between weeks 10 and 24 in women with missed abortions [19]. Similarly, Brew et al. (2007) reported that pregnant women with preeclampsia exhibited a smaller increase in DAO levels, accompanied by higher histamine concentrations in the placenta [44]. The authors suggested that some typical symptoms of preeclampsia, such as nausea, headache, hypertension, and edema, might be caused by histamine accumulation [44].

At two months postpartum, a reduction in DAO activity was observed, accompanied by a recurrence of symptoms, with an average of three symptoms per patient. However, the only symptom that worsened significantly after childbirth was migraine, whereas bloating, constipation, flatulence, rhinorrhea, and pruritus were significantly lower than before pregnancy. This suggests that the potential protective effect of increased DAO activity during pregnancy may persist for at least two months postpartum for these symptoms, except for migraine. It would have been valuable to have data on symptom progression beyond two months postpartum to determine whether the symptoms eventually returned to pre-pregnancy levels. Nevertheless, given the observed trend, implementing a specific dietary treatment postpartum to prevent symptom recurrence is advisable, even though the symptoms do not initially reappear at the same frequency.

This study also aimed to assess the prevalence of SNPs in the DAO-encoding gene (*AOC1*) in the participants. In agreement with the literature, rs10156191, rs1049793, and rs2052129 were the most common SNP variants identified [5,6,7,27]. Additionally, the frequencies of these variants were similar to those previously reported in non-intolerant populations and the European population, as documented in the ALFA database [5,6,27]. In line with these studies, no correlation was observed between the number of genetic variants and the number or type of symptoms [5,6,45].

In contrast, analysis of the relationship between serum DAO activity and SNP status revealed that patients carrying the rs1049793 variant in heterozygosity exhibited lower DAO activity, which aligns with the results of Ayuso et al. (2007) [7]. Similarly, Maintz et al. (2011) and Duelo et al. (2024) identified an association between reduced DAO activity and the rs2052129 variant in patients with histamine intolerance [5,6]. Despite these findings, it should be noted that the mere presence of an altered *AOC1* gene variant is insufficient as a standalone diagnostic marker for DAO deficiency, as no direct correlation was found between genetic variants and the number or type of symptoms in this population. Additionally, the five patients who were non-carriers of any altered SNPs may have presented with histamine intolerance of secondary intestinal origin, potentially resulting from impaired intestinal barrier function or other gut pathologies. In such cases, increased intestinal absorption of histamine into the bloodstream could trigger the appearance of symptoms, even in the absence of genetically determined DAO deficiency [3].

The main limitation of the present study was its relatively small sample size. This limited our ability to better assess the genetic influence within the studied population. Additionally, it would have been valuable to assess the intensity of clinical symptoms in patients at multiple time points during pregnancy and beyond two months postpartum using validated severity scales. Measuring DAO activity at additional key time points, such as weeks 20–22 and 36, would also help better characterize the dynamics between enzyme activity and symptom evolution.

## 5. Conclusions

In summary, this prospective observational study is the first to examine serum DAO levels and histamine intolerance symptoms in women before, during, and after pregnancy. The results revealed an improvement in histamine intolerance symptoms and an increase in serum DAO activity during pregnancy. Additionally, a decline in DAO activity was observed at two months postpartum compared to levels during pregnancy, accompanied by a mild recurrence of symptoms of histamine intolerance. The marked improvement in symptoms during pregnancy suggests that restrictive diets, such as low-histamine diets, may not be necessary for women with histamine intolerance during pregnancy.

## Figures and Tables

**Table 1:** Clinical baseline information of the participants of the study. Data are presented as mean ± standard deviation and (minimum–maximum).

Age (Year)	BMI *	Serum DAO Activity (U/mL)	Medical History
34.47 ± 4.84 (24–45)	20.51 ± 2.34 (17.40–26.20)	7.69 ± 5.20 (2.17–26.60)	27 patients did not present a relevant medical history 1 patient presented with insulin resistance # 2 patients presented with hypothyroidism #

* BMI: Body Mass Index. # To date, no recognized association has been reported between these two conditions and histamine intolerance.

**Table 2:** The frequency of symptoms associated with histamine intolerance recorded for all participants before, during (12–14 weeks), and two months after pregnancy.

Systems and Symptoms	Frequency (%) *			*p*-Value and Percentage (%) of Reduction (↓) or Increase (↑) of Women with Histamine Intolerance Symptoms		
	Pre-Pregnancy (A)	Pregnancy (B)	After-Pregnancy (C)	B vs. A	C vs. A	C vs. B
**Gastrointestinal tract**	**90**	**46**	**54**	**0.001 (49% ↓)**	**0.002 (40% ↓)**	0.564 (17% ↑)
Bloating	77	8	31	**<0.001 (90% ↓)**	**<0.001 (60% ↓)**	0.1 (287% ↑)
Constipation	43	27	19	0.077 (37% ↓)	**0.013 (56% ↓)**	0.480 (30% ↓)
Diarrhea	23	0	15	**0.014 (100% ↓)**	0.414 (35% ↓)	0.102 (-)
Flatulence	43	4	19	**<0.001 (91% ↓)**	**0.017 (56% ↓)**	0.174 (375% **↑**)
Vomiting	7	11	0	0.480 (57% ↑)	0.157 (100% ↓)	**0.034 (100% ↓)**
Reflux	23	4	0	**0.046 (83% ↓)**	**0.012 (100% ↓)**	0.617 (100% ↓)
Postprandial fullness	23	8	4	**0.041 (65% ↓)**	**0.041 (83% ↓)**	0.683 (50% ↓)
**Respiratory System**	**47**	**8**	**15**	**0.001 (83% ↓)**	**0.013 (68% ↓)**	0.480 (88% ↑)
Rhinorrhea	43	8	15	**0.003 (82% ↓)**	**0.027 (65% ↓)**	0.460 (88% ↑)
Sneezing	10	4	4	0.540 (60% ↓)	0.540 (60% ↓)	1 (-)
Asthma	0	0	0	-	-	-
**Skin**	**63**	**15**	**38**	**<0.001 (76% ↓)**	**0.014 (40% ↓)**	0.066 (153% ↑)
Pruritus	47	11	23	**<0.001 (77% ↓)**	**0.007 (51% ↓)**	0.245 (109% ↑)
Flushing	40	4	19	**<0.001 (90% ↓)**	**0.042 (53% ↓)**	0.174 (375% ↑)
Eczema	7	0	8	0.221 (100% ↓)	1 (14% ↑)	0.221 (-)
**Neurological system**	**80**	**15**	**54**	**<0.001 (81% ↓)**	0.055 (32% ↓)	**0.006 (260% ↑)**
Migraine ^#^	37	4	27	**0.002 (89% ↓)**	0.308 (27% ↓)	**0.042 (575% ↑)**
Headache	53	8	27	**<0.001 (85% ↓)**	**0.038 (49% ↓)**	0.137 (237% ↑)
Lack of concentration	7	4	0	0.480 (43% ↓)	0.157 (100% ↓)	0.480 (100% ↓)
**Circulatory system**	**50**	**4**	**11**	**<0.001 (92% ↓)**	**0.003 (78% ↓)**	0.460 (175% ↑)
Hypotonia	43	0	11	**<0.001 (100% ↓)**	**0.014 (74% ↓)**	0.221 (-)
Tachycardia	0	4	0	0.221 (-)	1 (-)	0.221 (100% ↓)
Dizziness	17	0	4	**0.014 (100% ↓)**	0.066 (76% ↓)	0.540 (-)
**Musculoskeletal** **system**	**77**	**19**	**54**	**<0.001 (75% ↓)**	0.137 (30% ↓)	**0.008 (184% ↑)**
Fatigue	63	19	38	**0.002 (70% ↓)**	0.126 (40% ↓)	0.126 (100% ↑)
Muscle pain	37	4	11	**0.002 (89% ↓)**	**0.020 (70% ↓)**	0.439 (175% ↑)

* The frequency (%) refers to the number of participants with these symptoms. ^#^ Patients with migraine were diagnosed by their respective neurologists. *p* < 0.05, marked in bold type, according to McNemar’s tests.

**Table 3:** DAO (*AOC1*) genotypes of women and pre-pregnancy serum DAO activity (mean ± SD) stratified by genotype groups.

SNP	Genotype	Patients (n = 30)	Patients (%)	DAO Activity (U/mL) Mean (SD)	*p*-Value
				7.69 (5.2)	
rs10156191	C/C ^#^	10	33.3	9.01 (6.82)	CC vs. CT 0.666
	C/T	16	53.3	7.65 (4.38)	CC vs. TT 0.088
	T/T	4	13.3	4.49 (2.19)	CT vs. TT 0.136
rs1049742	C/C ^#^	26	86.7	7.98 (6.35)	
	C/T	4	13.3	5.77 (2.35)	CC vs. CT 0.746
	T/T	0	0	-	
rs1049793	C/C ^#^	12	40	9.58 (4.48)	CC vs. CG 0.021
	C/G	15	50	6.44 (5.89)	CC vs. GG 0.799
	G/G	3	10	6.28 (0.53)	CG vs. GG 0.553
rs2052129	G/G ^#^	15	50	7.85 (5.77)	GG vs. GT 0.885
	G/T	13	43.3	7.79 (4.98)	GG vs. TT 0.721
	T/T	2	6.6	5.73 (2.87)	GT vs. TT 0.778

^#^ The most common allele frequency in the general population [27]. For example, in the rs10156191 gene variant, “CC vs. CT” refers to the comparison between the mean DAO activity of non-carrier patients and that of heterozygous carrier patients. Statistical significance was set at *p* < 0.05, determined using the Kruskal−Wallis test.

## Data Availability

The original contributions presented in this study are included in this article. Further inquiries should be directed to the corresponding authors.
